# Inhalation Pneumonitis Caused by Nebulized Hydrogen Peroxide

**DOI:** 10.7759/cureus.38116

**Published:** 2023-04-25

**Authors:** Andrew Manfra, Jill Sharma, Jeremy Kilburn

**Affiliations:** 1 Internal Medicine, Kirk Kerkorian School of Medicine at the University of Nevada, Las Vegas, Las Vegas, USA; 2 Pulmonary and Critical Care Medicine, University of Nevada, Las Vegas School of Medicine, Las Vegas, USA

**Keywords:** continuous positive airway pressure (cpap), lung toxicity, chemical pneumonitis, inhalation injury, hydrogen peroxide

## Abstract

Hydrogen peroxide is a chemical commonly used as a household antiseptic for cleaning and disinfecting. No cases of acute hydrogen peroxide inhalation-induced lung injury are previously described. We present a case of acute chemical pneumonitis caused by mixing hydrogen peroxide in a nighttime continuous positive airway pressure device's humidifier used for obstructive sleep apnea to prevent COVID-19 infection. The patient endorsed mixing hydrogen peroxide with distilled water in his nighttime continuous positive airway pressure device's humidifier at a ratio of 1:3-1:2 for the previous week before admission based on a friend's advice in preventing COVID-19. The presenting chest X-ray showed new multifocal consolidations with interstitial markings and alveolar edema throughout both lungs. Chest computed tomography (CT) imaging demonstrated multifocal, bilateral, hazy consolidations with increased interstitial markings and bilateral pleural effusions. The patient was subsequently initiated on systemic glucocorticoid therapy, significantly improving hypoxemia and dyspnea. Inhalation of hydrogen peroxide may produce acute pneumonitis distinct from what has been described previously with chronic inhalation. Given this case, systemic glucocorticoid therapy may be considered a viable treatment option for acute hydrogen peroxide-associated inhalation lung injury causing pneumonitis.

## Introduction

Chemical pneumonitis from inhalation requires the aspiration of toxic substances to the lower airways, independent of infection. It often presents with a history of hazardous exposure, such as industrial work. Pneumonitis caused by aspiration was initially described in 1946 by Mendelson in a study of aspirating gastric contents into anesthetized obstetric patients [1]. Cyanosis and respiratory distress soon developed within 2 hours. Despite the severity of initial symptoms, all patients had rapid recoveries within 24-36 hours. It is important to note that these were relatively healthy obstetric patients. Unfortunately, it is common to find multiple comorbidities in today's patient population, and such insults could be life-threatening.

However, in addition to gastric acid aspiration, many organic and inorganic compounds can cause chemical pneumonitis without aspiration. This includes medications such as the antiarrhythmic amiodarone [2], immunomodulators including the PD1/L1 inhibitor nivolumab3 [3], and chemotherapies such as bleomycin [4] and hydroxycarbamide [5].

Burn injuries from caustic agents typically lead to airway compromise requiring preemptive intubation due to fears of inflammatory swelling and bronchoconstriction causing sudden hypoxic respiratory failure [6]. The cough reflex impedes most insulting agents caused by inhalation to spare the lower airways from damage [7]. The initial imaging can be normal for up to 48 hours, with the most common radiographic finding being pulmonary edema; however, various radiographic opacities have been reported [8]. Inflammatory markers can be elevated, or normal and concurrent infections should always be considered. Long-term complications include bronchiectasis, bronchiolitis obliterans, and lung destruction [9]. In true inhalation chemical pneumonitis, treatments are generally supportive with avoidance of the insulting agent, and the evidence for glucocorticoids remains unclear [10-13]. We present a case of acute nebulized hydrogen peroxide inhalation injury causing chemical pneumonitis.

This case report was previously presented at the American Thoracic Society International Conference on May 14, 2021.

## Case presentation

**Figure 1:**
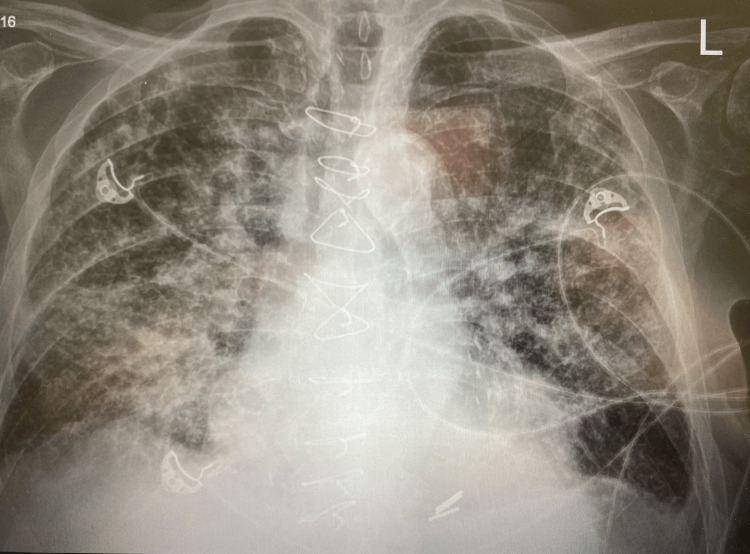
Chest X-ray showed new multifocal consolidations with interstitial markings and alveolar edema throughout both lung fields.

An 82-year-old male with a past medical history of diastolic heart failure, coronary artery disease with bypass grafting, obstructive sleep apnea, chronic obstructive pulmonary disease, and chronic kidney disease stage III presented to the emergency department with a chief complaint of dyspnea and intermittent morning hemoptysis. His symptoms started the week prior and had been progressively worsening. He denied increased sputum and endorsed a COVID-19 exposure two weeks earlier. His review of systems was significant only for dyspnea and morning hemoptysis. On presentation to the emergency department, he was tachypneic at rest with an oxygen saturation of 81% on room air. There was no evidence of sepsis as the patient was afebrile, without tachycardia, leukocytosis, or lactic acidosis. His only abnormal vital sign was tachypnea. The respiratory rate fluctuated between 20 and 30 respirations per minute. Physical exam was remarkable for increased respiratory effort and blood-tinged sputum. He received ceftriaxone 1g IV and doxycycline 100mg IV as a one-time dose on presentation to the emergency department, as well as 40mg IV furosemide and 60mg IV methylprednisolone. He was placed on supplemental oxygen with pulse oximetry, initially improving to 90% on 6 liters/min nasal cannula (Figure 1).

**Figure 2:**
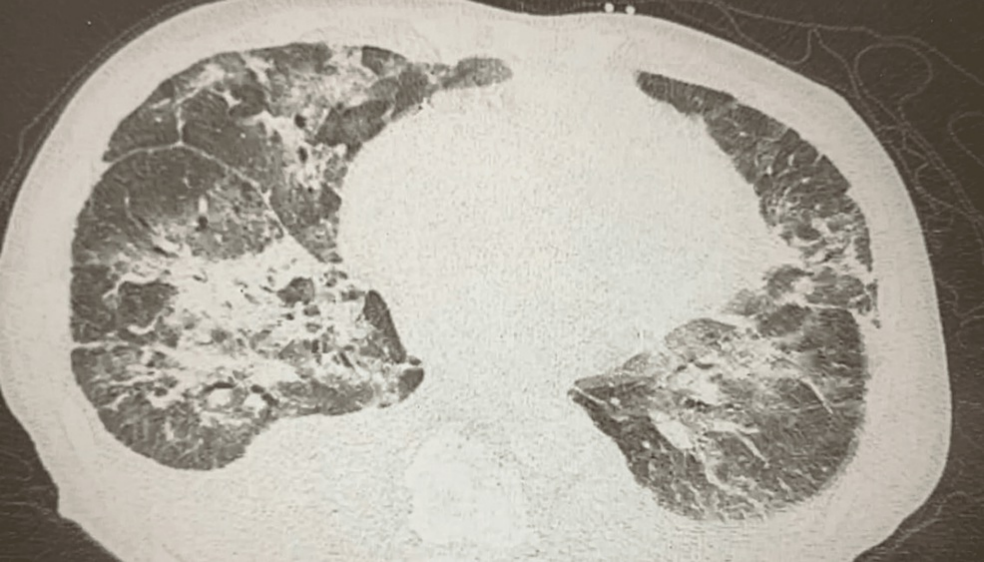
Chest CT imaging demonstrated multifocal, bilateral, hazy consolidations with increased interstitial markings and bilateral pleural effusions. CT: computed tomography

Pertinent lab results revealed negative viral respiratory polymerase chain reaction (PCR) testing and respiratory cultures with no growth of pathogenic bacteria. Inflammatory markers, including lactate dehydrogenase, erythrocyte sedimentation rate, c-reactive protein, d-dimer, and ferritin were elevated. The complete blood cell count showed no leukocytosis, and the comprehensive metabolic panel had no abnormalities. Autoimmune and fungal etiologies were ruled out via laboratory testing. Given clinical suspicion of COVID-19, antibody testing was ordered and resulted in negative. The B-type natriuretic peptide was 2826 ng/dL, the cardiac troponin's trend peaked at 0.3ng/dL, and the electrocardiogram showed normal sinus rhythm without ischemic changes. An echocardiogram on admission revealed new systolic dysfunction with a reduced ejection fraction of 35-40%. An echocardiogram three months previously showed an ejection fraction of 55-60% with impaired relaxation. He did not endorse any cardiac events that occurred since his previous echocardiogram. Cardiology was consulted, who recommended left heart catheterization after the resolution of his acute pneumonitis on an outpatient basis, given low concerns for acute coronary syndrome on presentation. The patient continued to have severe dyspnea and hypoxemia despite treatment for heart failure and chronic obstructive pulmonary disease consisting of diuretics and bronchodilators (Figure 2).

Peak oxygen requirements continued to increase, requiring a high flow nasal cannula at 50 liters/minute, 80% fraction of inspired oxygen (FiO2). Continuation of corticosteroids received in the emergency department was withheld pending the finalization of the infectious workup consisting of blood and respiratory cultures. The patient deferred diagnostic bronchoscopy as he was not agreeable to intubation. The patient did not report a history of hydrogen peroxide inhalation until day three of admission after oxygen requirements continued to increase, and there was concern that the patient may go into cardiopulmonary arrest. When discussing goals of care and concerns for decompensation, the patient admitted that he was nebulizing hydrogen peroxide to prevent COVID-19. The patient recounted mixing hydrogen peroxide in his nighttime continuous positive airway pressure device's humidifier at a ratio of 1:2-1:3 with distilled water for the previous week before admission. This was based on a friend's advice in preventing COVID-19. The patient was subsequently initiated on 1mg/kg methylprednisolone twice daily for three days and showed significant improvement in hypoxemia with oxygen requirements decreasing to the nasal cannula. He has transitioned to a two-week prednisone taper with oxygen requirements decreased to 2 liters/min nasal cannula at the time of discharge. The discharge diagnosis was inhalation chemical pneumonitis from hydrogen peroxide-induced lung injury and new onset heart failure with reduced ejection fraction. The patient was seen in the pulmonology clinic on a nursing visit 21 days after hospital discharge to assess oxygenation status. His pulse oximetry on room air was spO2 94%. His pulse oximetry with ambulation was spO2 90%, and home oxygen therapy was discontinued.

## Discussion

This case demonstrates a rare acute inhalation pneumonitis after the recent use of hydrogen peroxide in a nighttime continuous positive pressure airway device's humidifier. Hydrogen peroxide is a chemical commonly used as a household antiseptic for cleaning and disinfecting. In a literature review, no acute hydrogen peroxide inhalation-induced lung injury cases have been described in humans. Due to influential media attention and fear, cleaning products such as bleach received disproportionate attention in the killing of the COVID-19 virus during the pandemic. Similarly, hydrogen peroxide is used as a disinfectant, which was intentionally inhaled in large amounts and over a one-week duration in this case.

Hydrogen peroxide has been noted to cause toxicity via three mechanisms, including corrosive damage, oxygen gas formation, and lipid peroxidation [14]. The effects of hydrogen peroxide on respiratory cilia and viability via direct exposure in mouse models have shown significant cytotoxic effects on ciliated respiratory epithelial cells by promoting cell death [15]. In vitro, the effects of hydrogen peroxide on ciliated epithelium showed significant cilial slowing and epithelial damage [16]. In Guinee pig models, hydrogen peroxide inhalation led to intense inflammation related to the suspected generation of hydroxyl radicals [17].

Prior case reports on hydrogen peroxide-associated lung injury in humans describe environmental and occupational exposure over 3-5 years, developing into a non-specific interstitial pneumonia pattern [18]. Chronic inhalation of hydrogen peroxide is described as causing interstitial lung disease, with radiographic images demonstrating septal line thickening, honeycombing, and traction bronchiectasis with associated ground glass opacification [19]. In an observational study of dental clinic workers that use dry fogging of hydrogen peroxide to disinfect surfaces, some developed symptoms, including dyspnea, cough, and nasal burning at an average hydrogen peroxide concentration of 1.3 to 2.83 parts per million in room air [20]. Similarly, in our case, the presenting symptoms were dyspnea and cough; however, our case demonstrates a much larger exposure given direct nebulization.

It remains unclear if the patient's new onset systolic heart failure was related to the nebulization of hydrogen peroxide. His presenting symptoms, cardiac troponins, and electrocardiogram were not consistent with myocardial infarction. The echocardiogram performed on admission compared to the prior three months showed a significant decrease in systolic function. He did not endorse any cardiac events or hospital admissions to suspect a recent myocardial infarct. Without a left heart catheterization, we cannot say conclusively what caused his worsening ejection fraction. Cardiology deferred this as it was considered non-emergent and could be completed by cardiology in the outpatient setting after the resolution of his pulmonary insult. It is possible that the nebulization of hydrogen peroxide precipitated a coronary artery bypass graft failure sometime the week before the presentation. However, there was no evidence that this happened acutely on presentation.

Systemic glucocorticoid therapy was initiated after the history revealed that the inhalation of nebulized hydrogen peroxide was driving the patients worsening respiratory status. The lung imaging was suspected to be consistent with an acute pulmonary insult related to the toxic inhalation of hydrogen peroxide. Infectious and autoimmune etiologies were ruled out via laboratory testing, and the patient history led to the diagnosis of acute inhalation-induced chemical pneumonitis. Given the novelty of this pulmonary insult and response to glucocorticoid therapy, this provides a basis for considering systemic glucocorticoids as a therapeutic treatment option in acute toxic inhalation injury secondary to nebulized hydrogen peroxide.

## Conclusions

Inhalation of hydrogen peroxide may produce acute pneumonitis distinct from what has been described previously with chronic inhalation. Patients may present with intermittent hemoptysis and cough associated with dyspnea without systemic symptoms. Inflammatory markers may be elevated in the absence of other significant lab work abnormalities. Given the novelty of this pulmonary insult and response to glucocorticoid therapy, this provides a basis for considering systemic glucocorticoids as a therapeutic treatment option in acute toxic inhalation injury secondary to nebulized hydrogen peroxide.
